# Environmental heterogeneity determines beta diversity and species turnover for woody plants along an elevation gradient in subtropical forests of China

**DOI:** 10.48130/FR-2023-0026

**Published:** 2023-10-31

**Authors:** Lan Jiang, Xin Zhang, Jing Zhu, Xin Wei, Bo Chen, Jinfu Liu, Shiqun Zheng, Zhongsheng He

**Affiliations:** 1 College of Forestry, Fujian Agriculture and Forestry University, Fuzhou City, Fujian 350000, China; 2 Key Lab of Ecology and Resources Statistics of Fujian Colleges, Fuzhou City, Fujian 30000, China; 3 Institute of Microbiology, School of Ecology and Nature Conservation, Beijing Forestry University, Beijing 100091, China

**Keywords:** Beta diversity, Species turnover, Species nestedness, Elevation gradient, Community assembly, Environmental filtering

## Abstract

To understand how diversity change with environmental gradients is a fundamental aim for clarifying biodiversity pattern and underlying mechanisms. Here, we studied the characteristics of beta diversity and its partitioning components for woody plant communities along an elevation gradient in subtropical forests of China, and thus explored the effects of environment and space on beta diversity. By using the Classification Method, we divided the species of Daiyun Mountain into four groups, namely generalists, high-elevation specialists, low-elevation specialists and rare species. We then calculated beta diversity, and partitioned it into species turnover and species nestedness. dbRDA was conducted to analyze the impact of spatial and environmental distance on the beta diversity and its partitioning components. Beta diversity comprised of two components: species turnover and species nestedness. Species turnover was the larger contributor to total beta diversity, and it tended to increase as elevation changed. This pattern can be attributed to environmental heterogeneity, resulting in the differentiation of specialized species and an increase in species turnover and beta diversity. Environmental factors, specifically the air temperature and slope, played a significant role in explaining the variation of turnover and beta diversity. However, spatial variables did not have a significant influence on these patterns. The maintenance of biodiversity in Daiyun Mountain was mainly governed by environmental filtering. Future conservation efforts should focus on strengthening the protection of specialized species in high elevation areas.

## Introduction

Beta diversity is an important spatial measure of biodiversity that assesses the differences in species composition among different communities across habitat gradients^[1]^. It is used to quantify the species composition differences among various environmental gradients or regions. Beta diversity not only reflects the pattern of regional biodiversity, but also provides insight into the relationship between species and the environment, which can be used to infer the processes underlying community assembly. Community assembly can be explained by two theories, niche theory and neutral theory^[2,3]^. Niche theory predicts that environmental variables are the main factors influencing species diversity patterns, while neutral theory suggests that spatial distance better explains species diversity patterns^[4−6]^. The spatial pattern of beta diversity is influenced by both environmental filtering and dispersal limitation, although their respective contributions differ. Therefore, understanding the underlying mechanisms that drives the variation of beta diversity is of great significance^[7,8]^.

A variety of methods have been proposed to measure beta diversity, which has expanded the application of beta diversity in ecology and conservation biology^[1,9,10]^. Among these methods, the Jaccard and Sørensen dissimilarity index are widely utilized^[11−13]^. Baselga was the first to propose a method of beta diversity partitioning based on the Sørensen index. This method can separate beta diversity into two components: species turnover and species nestedness^[14]^. Species turnover refers to the replacement of species between communities, which may be a result of niche differentiation, evolutionary processes or dispersal limitation^[15,16]^. Species nestedness reflects changes in species richness caused by species loss or gain, often related to historical processes such as selective extinction and selective colonization^[17]^. Most studies have consistently indicated that turnover is the primary component of beta diversity^[18−20]^. This implies that the ecological processes shaping the spatial distribution of beta diversity can be approached from the perspective of turnover. Habitat heterogeneity hypothesis suggests that greater heterogeneity is more likely to have a greater species occupancy, leading to higher species diversity and more specialized species^[21,22]^. This, in turn, contributes to greater community turnover and beta diversity^[23]^. Dispersal limitation also contributes to turnover by promoting species aggregation^[24]^, with lower dispersal capacity associated with higher species turnover and beta diversity^[16]^. Both environmental filtering and dispersal limitation play a crucial role in influencing species range size, consequently changing the balance between specialized and generalized species within a community. These factors have an impact on species turnover and, ultimately, the level of beta diversity within a community^[25,26]^. Investigating the relationship between beta diversity and different types of species enable deeper exploration of the potential causes of community spatial variation^[6]^.

High habitat heterogeneity along the environmental gradient provides an ideal platform for the study of beta diversity and its component decomposition^[18,27,28]^. However, previous research has primarily focused on the latitudinal gradient, there has been relatively limited research on the elevation gradients in mountains. Mountains only account for 25% of the total area of terrestrial ecosystems on earth, but they conserve more than 85% of the global biological taxa^[29]^. The elevation gradients in mountains lead to extreme environmental variations over relatively short distances. For instance, temperature can change 1,000 times faster in mountains compared to latitudinal gradients^[30]^. McFadden et al.^[27]^ has shown that these rapid environmental changes in mountains contribute to a higher variation in beta diversity compared to local regions, with the environmental influence on beta diversity of mountain communities being more pronounced. Mountains therefore provide natural experimental sites for studying community and ecosystem processes, making them crucial areas for biodiversity research and conservation^[31]^. Currently, research on beta diversity along elevation gradients primarily focus on the variation in diversity of different taxa^[28]^, the distribution pattern of beta diversity^[20,32,33]^, the partitioning of beta diversity^[34−36]^, and the separation of spatial and environmental effects^[33,37]^. There exists a certain relationship between species, beta diversity, turnover, and the environment. Integrating these factors is crucial for gaining a comprehensive understanding of beta diversity.

Daiyun Mountain, located in the transitional zone between central and south tropical regions, has a large elevation change where the highest elevation reaches 1,856 m. With the increase of elevation, mountain evergreen broad-leaved forest (700−1,000 m), coniferous‐broadleaf mixed forest (1,000−1,400 m), coniferous forest (1,400−1,650 m) and moss pygmy forest (> 1,650 m) are distributed successively, forming a natural vertical vegetation spectrum^[38]^. Research on biodiversity in Daiyun Mountain has primarily focused on α diversity and phylogenetic diversity^[39]^. However, limited studies have been conducted on beta diversity. Understanding beta diversity and its partitioning components along elevational gradients is crucial for comprehending the mechanisms behind changes in species composition^[40]^. Thus, our study aimed to investigate the elevational pattern of beta diversity and its partitioning components, and analyze the influence of spatial and environmental factors on beta diversity and its components. Specifically, we sought to answer the following three questions: (1) How do beta diversity and its partitioning components vary along the elevation gradient in Daiyun Mountain? (2) What are the relative contributions of environmental filtering and dispersal limitation to the distribution pattern of beta diversity? (3) What is the relationship between environment, species, beta diversity and its decomposing components?

## Materials and methods

### Study area

Daiyun Mountain National Nature Reserve (25°38'07″−25°43'40″N, 118°05'22″−118°20'15″N) is located in Dehua County, Fujian Province, China. With a maximum elevation of 1,856 m, it exemplifies a typical coastal mountainous forest ecosystem in southeastern China. The region boasts diverse topography, noticeable elevation gradients, moderate temperatures, and a distinctive microclimate. The predominant vegetation type is subtropical evergreen broad-leaved forest, while the primary soil type is mountain Ferric acrisols soil^[41]^.

### Data collection

**Figure 1:**
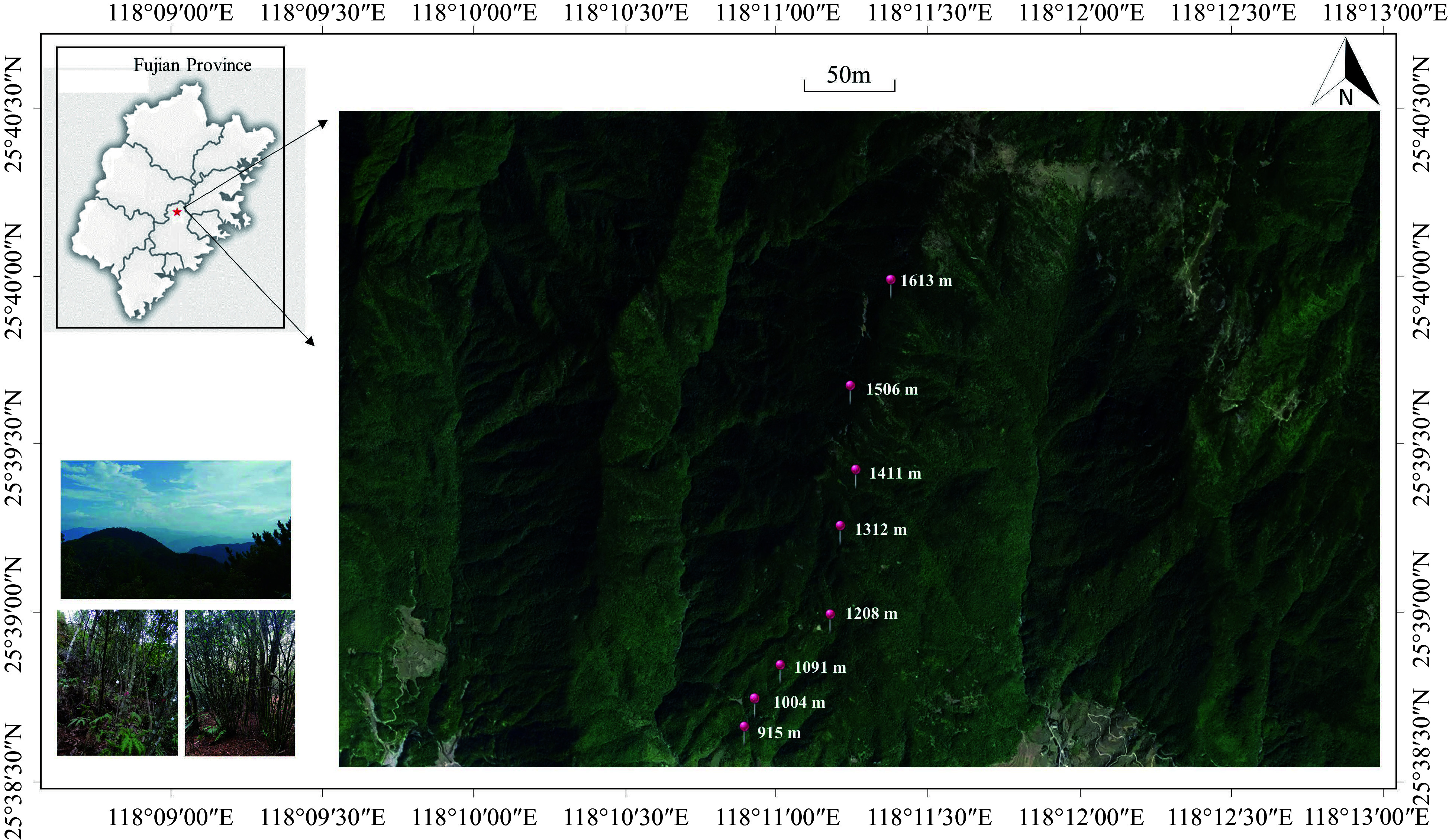
Distribution of sampling plots along the elevation gradient in Daiyun Mountain.

Eight 20 m × 30 m plant community sampling plots were set at intervals of 100 m at an elevation of 900−1,600 m of Daiyun Mountain. Each plot was further divided into three 10 m × 20 m quadrats (see Fig. 1). All woody plants within the quadrats with DBH ≥ 1 cm were identified for species by *Flora of China* (http://www.iplant.cn/foc), the abundance was recorded, and the DBH and height were measured.

Longitude, latitude and elevation are regarded as spatial factors. Environmental factors can be broadly categorized into three groups: topographic factors, climate factors and soil factors. Topographic factors include slope and aspect. Climate factors include soil temperature and air temperature. Soil factors include soil water content, total carbon, total nitrogen, total phosphorus, total potassium, soil pH, hydrolyzed nitrogen and available phosphorus content. For detailed information on environmental factors measured refer to Chen et al.^[41]^.

### Data analysis

#### Community species composition

Species composition of each community was characterized by species abundance, richness and importance value. The importance value was evaluated by the sum of species relative abundance, frequency and dominance. The calculation details of importance value are as per the methods of Jiang et al.^[42]^.

#### Classification of community species

To investigate the distribution pattern of different types of species along elevational gradients, species were categorized into generalists (species with a widespread distribution range) and specialists (species with a restricted distribution range), based on their abundance and distribution range. The two-habitat approach of CLAM (Classification Method) is well-suited for classifying the generalists and specialists within compared habitats^[43]^. By using CLAM, species along the elevation gradient in Daiyun Mountain could be classified into four groups: generalists, high-elevation specialists, low-elevation specialists, and rare species. Generalists refers to species distributed at two elevational communities, such as *Adinandra millettii*, which is abundant at both elevations of 900 m and 1,000 m. Specialists, on the other hand, have a high distribution in one community but little or no distribution in another. Based on the relative elevation of the two communities, high-elevation specialists and low-elevation specialists were distinguished. For example, *Castanopsis faberi* is distributed at an elevation of 900 m, but not at an elevation of 1,000 m, and therefore, it is considered a low-elevation specialist. Conversely, *Symplocos stellaris* is mainly distributed at an elevation of 1,000 m, with only one individual found at an elevation of 900 m, indicating it is a high-elevation specialist. Lastly, rare species are species that are too rare to be classified within a specific community. The advantage of this model is that it can increase the number of habitat specialists, which is highly consistent with other methods.

There are two main influencing parameters. One is the *K* value, which determines whether the threshold is strict or not. It is recommends to select *K* = 2/3 for small samples^[43]^. The other is *p* value to test whether the classification is significant, which can be 0.05, 0.01, 0.005 or 0.001. Affected by the number of tests, the *p* value should be adjusted, and *p *= 0.005 or 0.001 is recommended. To classify species in Daiyun Mountain, we selected a super-majority threshold of *K *= 2/3 and *p* = 0.005. Community species classification was analyzed by the CLAM program (http://purl.oclc.org/clam).

To investigate the difference in species composition with increasing elevation change, a linear regression analysis was conducted. The elevation change was used as the explanatory variable, while the number of species from CLAM served as the response variable.

### Beta diversity and its partitioning component

Beta diversity along the elevation gradient in Daiyun Mountain was calculated using Sørensen heterogeneity and Bray-Curtis indices based on presence-absence data and abundance data, respectively. To determine the source of species composition between paired communities, total beta diversity based on presence-absence data (β_sor_) was partitioned into components of turnover (β_sim_) and nestedness (β_sne_)^[14]^. Similarly, the total beta diversity based on abundance data (d_BC_) was partitioned into balanced changes in species abundances(d_BC-bal_) and abundance gradients(d_BC-gra_)^[35]^. The data analysis was computed using the *betapart* package in R 4.0.3^[36]^.

A regression model was employed to analyze the variation of beta diversity and its partitioning components with increasing elevation change. Both simple linear regression and binomial regression were found to better fit the relationship between elevation change and beta diversity. The goodness of fit of the two models was compared using the 'anova' function. The results indicated that for beta diversity and its partitioning component with presence-absence data, linear regression was the better choice. For beta and turnover with abundance data, binomial models provided a better fit, while linear models were more suitable for nestedness (Supplemental Table S1).

### Spatial and environmental influence

Firstly, the horizontal position is determined utilizing PCNM, which quantifies the spatial arrangement of sample units by calculating principal coordinates based on a truncated distance matrix^[33]^. The PCNM axes with positive eigenvalues were subsequently used as spatial explanatory variables in constraint ordination analysis. Environmental variables considered included air temperature, slope, slope aspect, soil water content, soil temperature, total carbon, total nitrogen, total phosphorus, soil pH, hydrolyzed nitrogen and available phosphorus content. Secondly, forward selection based on 999 permutations was used to identify the spatial and environmental variables with significant influence as explanatory variables. Among these variables, only air temperature (AT) and slope (SLOP) were retained (Supplemental Table S2), while spatial factors were found to have no significant influence (Supplemental Table S3). Before the forward selection, clustering method of *Hmisc* package was used to evaluate multicollinearity relationship among explanatory variables, and ten explanatory variables were retained (Supplemental Fig. S1). Finally, the logarithm of air temperature and slope were used to analyze the environmental effect on beta diversity and its components using the method of dbRDA. This analysis was conducted by *vegan* package in R 4.2.3.

## Results

### Community species composition along the elevation gradient in Daiyun Mountain

A total of 5,926 woody plants individuals with the total of 91 species were found along the elevation gradient in Daiyun Mountain. *Cyclobalanopsis glauca* or *Cunninghamia lanceolata* was the dominant species at lower elevation (Table 1). The dominant species at the middle elevation were mainly *C. lanceolata*, *Machilus thunbergii*, *Eurya rubiginosa* var. *attenuata* and *Pinus taiwanensis*, while the *P. taiwanensis* was the dominant species at higher elevation.

**Table 1:** Community structure and species composition of wood plant community in Daiyun Mountain.

Plots	Abundance	Species richness	Dominant species (importance value)
DYS900	682	33	*Cyclobalanopsis glauca* (34.816)
DYS1000	509	35	*Cunninghamia lanceolata* (34.672)
DYS1100	432	31	*C. lanceolata* (33.510)
DYS1200	333	39	*C. lanceolata* (17.609); *Machilus thunbergii* (14.473)
DYS1300	809	32	*Eurya rubiginosa* var. *attenuata* (16.141); *Pinus taiwanensis* (15.129)
DYS1400	1,007	31	*P*. *taiwanensis* (27.780)
DYS1500	864	25	*P*. *taiwanensis* (32.591)
DYS1600	1,290	24	*P*. *taiwanensis* (31.874)

### Changes of generalists, specialists and rare species at different elevations

**Figure 2:**
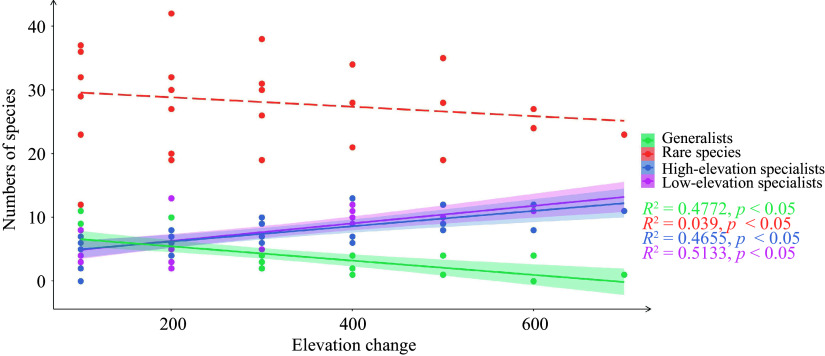
Community species composition with the increase of elevation changes. The elevation changes refer to the vertical difference in height between two paired elevations.

With the increasing elevation change, the number of generalists significantly decreased, while the number of specialists increased gradually (Fig. 2). The number of rare species was consistently highest at each elevation, but this was not significantly correlated with the elevation change.

### Beta diversity and its partitioning components

**Figure 3:**
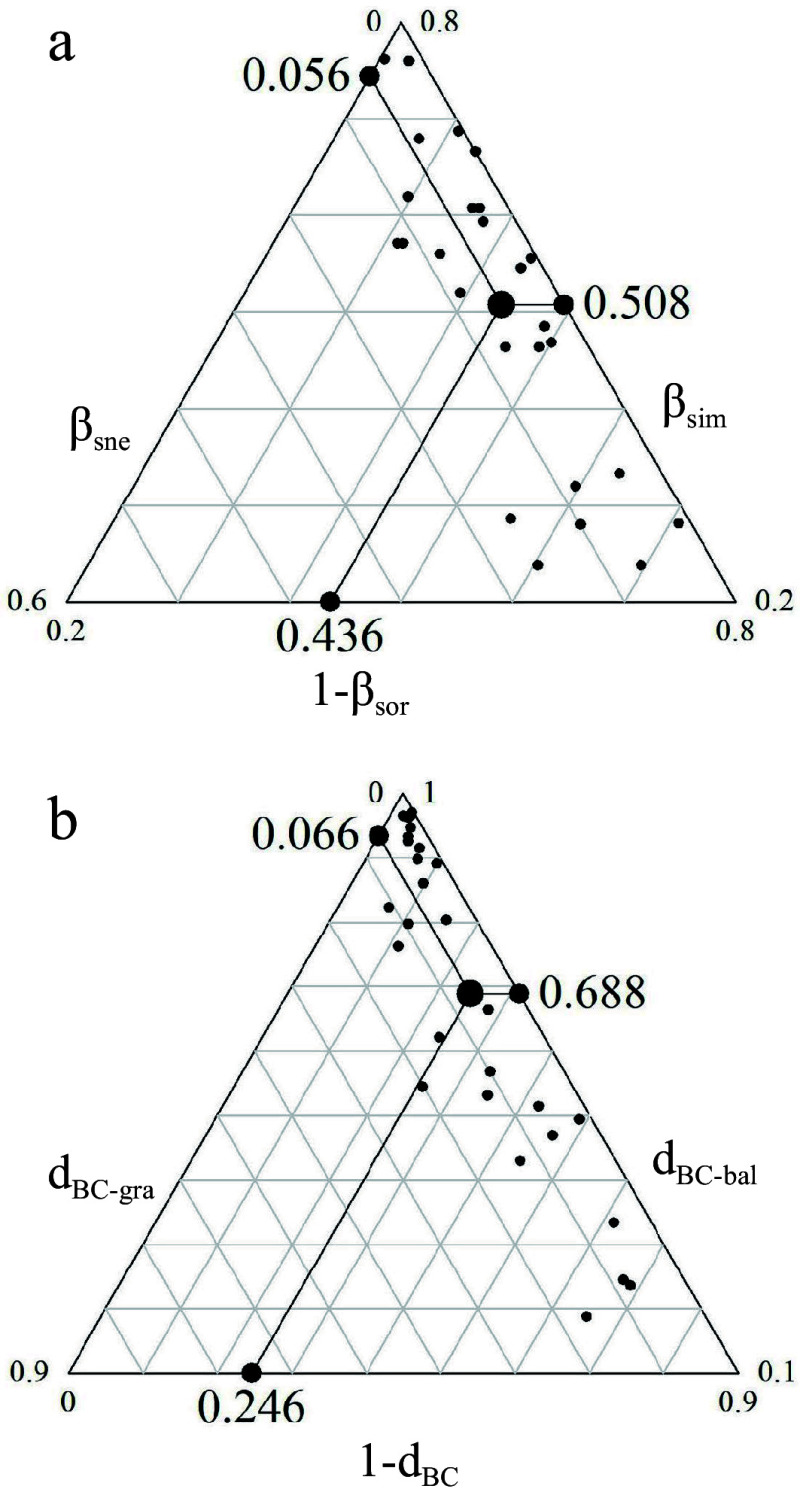
Community beta diversity and its components in Daiyun Mountain. (a) Displays the beta diversity (β_sor_), species turnover (β_sim_), and species nestedness (β_sne_) using presence-absence data. (b) Presents the Bray-Curtis distance (d_BC_), balanced variation (d_BC-bal_), and abundance gradient (d_BC-gra_) using abundance data.

Beta diversity in Daiyun Mountain, as measured using presence-abundance data and abundance data, was found to be 0.564 and 0.754, respectively (Fig. 3). The beta diversity and turnover obtained from abundance data were higher than those obtained from presence-abundance data. Regardless of the type of data used, turnover was identified as the major component to beta diversity.

**Figure 4:**
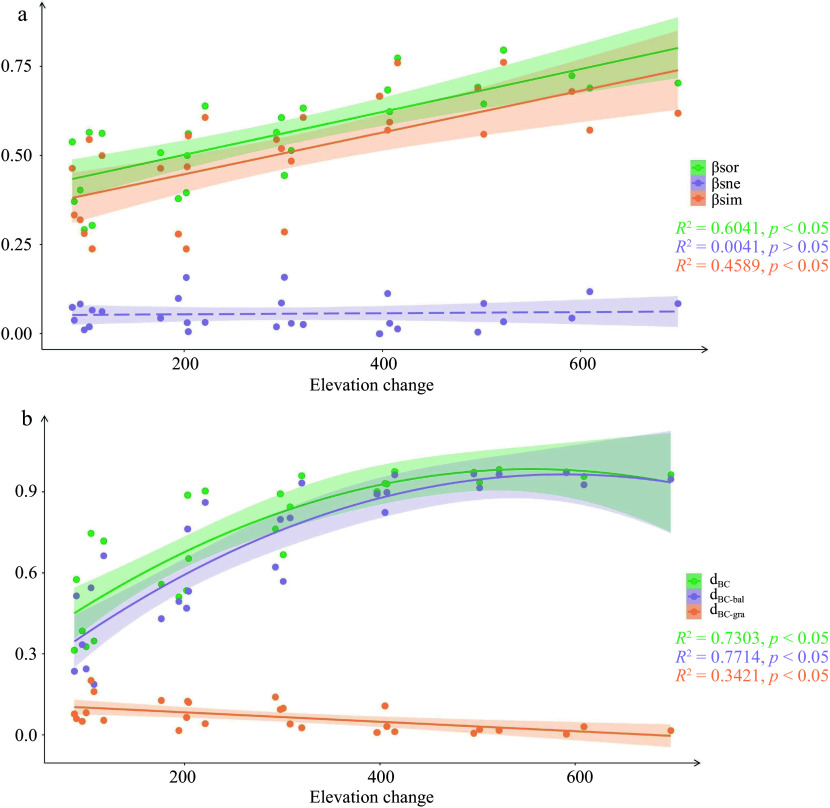
Community beta diversity and its components changed with the increase of elevation change. (a) Presence-absence data. (b) Abundance data.

Both beta diversity and turnover increased with elevation change, with significantly higher values at higher elevations compared to lower elevations (Fig. 4). However, there were differences in beta diversity and turnover between presence-absence data and abundance data. Beta diversity and turnover, when measured using presence-absence data, increased linearly with elevation change. In contrast, when measured using abundance data, beta diversity and turnover showed a significant quadratic regression as elevation change increased. With respect to nestedness components, as elevation change increased, there was a significant decrease in nestedness based on abundance data. However, there was no significant distribution pattern observed between nestedness based on presence-absence data and increasing elevation change.

### Influence of spatial and environmental distance on beta diversity

Spatial variables had no significant effect on species distribution in Daiyun Mountain (Supplemental Table S3). During the forward selection process, only AT and SLOP of environmental variables were retained (Supplemental Table S2). Environmental variables explained 56.0% and 64.7% of the variation in beta diversity (Table 2), as indicated by presence-absence data and abundance data, respectively. Among the environmental variables, air temperature emerged as the most important factor in shaping beta diversity and turnover. When considering the full model including air temperature and slope, the nestedness components did not show any significant effect. However, the independent model that only included slope showed a significant effect on nestedness.

**Table 2:** dbRDA analysis based on presence-absence data and abundance data.

Dataset	Responsible variable	Model	*R* ^2^	*p*
Presence-absence	β_sor_	~AT+SLOP	0.560	0.006
		AT	0.923	0.002
		SLOP	0.601	0.097
	β_sim_	~AT+SLOP	0.565	0.005
		AT	0.883	0.008
		SLOP	0.657	0.066
	β_sne_	~AT+SLOP	0.178	0.950
		AT	0.280	0.417
		SLOP	0.916	0.001
Abundance	d_BC_	~AT+SLOP	0.647	0.002
		AT	0.884	0.005
		SLOP	0.782	0.017
	d_BC-bal_	~AT+SLOP	0.669	0.004
		AT	0.838	0.010
		SLOP	0.602	0.106
	d_BC-gra_	~AT+SLOP	0.228	0.872
		AT	0.143	0.690
		SLOP	0.860	0.009
AT and SLOP are air temperature and slope, respectively.				

## Discussion

### Species composition along elevational gradients

The species composition of woody plants in Daiyun Mountain exhibited significant variation along the elevation gradient, with a noticeable change in dominant species occurring between elevations of 1,200 m and 1,300 m. Below an elevation of 1,200 m, the dominant species was *C. lanceolate*, whereas above 1,300 m it transformed into *P. taiwanensis*. This transition zone also marks the upper limit of the coniferous-broadleaf mixed forest, as the vegetation types above 1,300 m transform into coniferous forests dominated by *P. taiwanensis*. Furthermore, the highest of beta diversity of neighboring elevations was observed at elevations between 1,200−1,300 m (Supplemental Fig. S2), which means that this area had the largest difference of community species composition. It can be inferred that the elevation range of 1,200−1,300 m serves as a transition zone for species distribution along the elevation gradient, where many species meet and replacement, resulting in the peak of species richness. This was supported by the study of Li et al.^[39]^, which found that species diversity showed a unimodal pattern, peaking at an elevation of 1,200 m.

The composition of generalists and specialists was found to undergo significant changes with elevation change increase. Notably, there was a significant reduction in the number of generalists, whereas the number of specialists experienced a significant increase. These findings suggest that as elevation change increases, community species composition tends to shift towards an increased abundance of specialists and a reduced abundance of generalists. Compared to specialized species, generalized species exhibit a broader ecological tolerance and a wider distribution range^[44]^. As a result, an increase in the number of generalists results in a greater number of shared species among communities. In contrast, specialists have a narrow ecological tolerance, and the variations in environmental conditions along elevation gradients contribute to the differentiation of specialists and increase the dissimilarity between paired communities^[20, 45]^. The introduction of specialized species leads to an increase in beta diversity, whereas the introduction of generalized species is associated with a decrease in beta diversity^[27]^.

### Beta diversity and its partitioning components

In Daiyun Mountain, the distribution pattern of species beta diversity along the elevational gradient was predominantly composed of species turnover, while nestedness contributed only a small portion. This finding aligns with the beta diversity and its components observed in the majority of previous studies^[23, 28, 45]^. Both abundance data and presence-absence data revealed a dominant role of turnover component, indicating that variation of species composition between communities in Daiyun Mountain involved both species turnover and abundance changes. The turnover of species between neighboring pairs of communities was influenced by both generalists and specialists, while turnover between distant communities was primarily driven by specialists (Fig. 2). Changes in species abundance also contributed to the shift in species dominance. For instance, the importance value of *C. lanceolata* gradually declined from an elevation of 1,000 m to 1,200 m, while the importance value of *P. taiwanensis* gradually increased from an elevation of 1,300 m to 1,600 m. Overall, the changes in species composition along elevation gradient were primarily driven by species turnover and abundance changes.

The beta diversity and turnover were found to increase with elevation change, which was consistent with previous studies^[46, 47]^. Environmental factors were found to have a primary influence on the distribution patterns of beta diversity and turnover, explaining 56.0% and 64.7% of the variation based on presence-absence data and abundance data, respectively. These findings suggest that environment filtering dominates community assembly in Daiyun Mountain, with niche processes playing a significant role. With the increase in elevation change, there was a corresponding increase in environmental heterogeneity. This increased heterogeneity leads to environmental filtering, where species are constrained to suitable habitats, resulting in alterations to their range size^[19, 48]^. Consequently, this process promotes the differentiation of specialized species, leading to heightened species turnover and ultimately enhancing beta diversity^[44, 45]^. Among the environmental factors considered, air temperature and slope are found to be the most important factors driving changes in beta diversity and turnover. Air temperature plays a crucial role in species distribution due to its impact on the thermal tolerance of organisms^[49]^. In particular, the harsher climate in higher elevations enhances species' sensitivity to temperature, consequently affecting their elevational distribution^[18]^. On the other hand, slope, as a topographic factor, primarily influences species distribution by altering topographic and soil nutrient conditions^[50]^. The combined influence of these climatic and topographic factors drives the distribution of species diversity along the elevation gradient in Daiyun Mountain.

Spatial variables also play a crucial role in shaping the distribution pattern of beta diversity, particularly in stochastic processes that are dominated by dispersal limitation. Qian ^[16]^ demonstrated that spatial distance primarily accounts for the variation in beta diversity, indicating a negative relationship between beta diversity and dispersal ability. In the case of mountains of temperate forest in China, Yu et al. have found that spatial distance has a greater impact than environmental factors in explaining beta diversity^[51]^. However, spatial factors did not have a significant impact on beta diversity in Daiyun Mountain. This could be attributed to the limited horizontal spatial extent of the sampling plots in Daiyun Mountain, which may not be sufficient to observe the distance-decay effect on beta diversity. On a finer scale, the importance of environmental factors outweighs that of spatial factors^[52]^. Additionally, the sampling plots in Daiyun Mountain were strategically placed along the ridgelines, leading to a strong spatial connectivity. This allows species to disperse smoothly to suitable habitats, minimizing the influence of spatial factors on turnover and beta diversity of Daiyun Mountain. Consequently, studies focusing on local-scale biodiversity highlight the influence of environmental impacts.

### Comparison of abundance and presence-absence data

Compared to presence-absence data, the use of abundance data resulted in higher values of beta diversity and turnover. Furthermore, a significant linear relationship between nestedness and elevation change was observed, along with a higher model *R*^*2*^ for environmental variables in db-RDA. Presence-absence data are recognized for higher sensitivity to rare species, often leading to an overestimation of beta-diversity^[53]^. As a result, presence-absence data is not suitable for research conducted in a small area with a higher abundance of rare species^[54]^. In contrast, abundance data provides a more comprehensive information of community and is more robust in cases of inadequate samplings^[55, 56]^. Therefore, it is advisable to prioritize the use of abundance data in beta diversity.

### Guides on biodiversity conservation

In terms of conserving species diversity, the difference in species turnover and species nestedness components has implications for conservation measures^[57]^. When nestedness is dominant, conservation planning should prioritize regions with higher species richness^[34, 57]^. When communities are primarily characterized by species turnover, multi-regional conservation becomes important^[34, 57]^. However, the dominant role of turnover is widely recognized, and implementing a multi-regional conservation strategy can complicate conservation efforts significantly. Cordeiro et al. suggested that in cases where beta diversity is mainly determined by turnover, species-poor communities do not simply represent a subset of species-rich communities^[23]^. Instead, these communities exhibit distinct species compositions across different sites. As a result, when turnover is the primary factor, species-poor sites hold greater conservation value than species-rich sites. Expanding on this perspective, the preservation of biodiversity in Daiyun Mountain should prioritize the high elevation region. Specifically, specialized species plays a key role in species turnover and beta diversity. These species tend to have restricted distributions, smaller populations, and limited phenotypic plasticity, making them less adaptable to environmental changes^[58,59]^. As a result, specialized species are more vulnerable to extinction in a changed environment^[60]^. Therefore, conservation of specialized species becomes a primary objective in maintaining biodiversity. In this context, specialized species at high elevation deserve special attention.

## Conclusions

The plant community in Daiyun Mountain displayed significant variation in turnover and beta diversity. With the increase of elevation change, the environment becomes more heterogeneous, leading to differentiation of specialized species and consequently an increase in species turnover and beta diversity. The variation in turnover and beta diversity is mainly explained by environmental factors, with no significant influence from spatial variables. Specifically, air temperature and slope were identified as the main factors affecting community assembly of woody plants in Daiyun Mountain. This suggested that community assembly in the area was predominantly governed by environmental filtering. To conserve biodiversity in Daiyun Mountain, it is important to focus on specialized species and prioritize the protection of high elevation areas. In future, efforts should be directed towards strengthening the protection of specialized species in these high elevation areas.

## SUPPLEMENTARY DATA

Supplementary data to this article can be found online.

## Data Availability

The datasets used and/or analyzed during the current study are available from the corresponding author on reasonable request.
