# Supplementary material for: Environmental heterogeneity determines beta diversity and species turnover for woody plants along an elevation gradient in subtropical forests of China
Source: For Res (Fayettev). 2023 Oct 31;3:26. doi: 10.48130/FR-2023-0026 (PMC11524245; doi:10.48130/FR-2023-0026)
Supplement: Supplementary file 1 — Supplementary data to this article can be found online. [file FR-2023-0026-S1.zip › 10.48130_FR-2023-0026-Suppl-FigureS2.pdf]

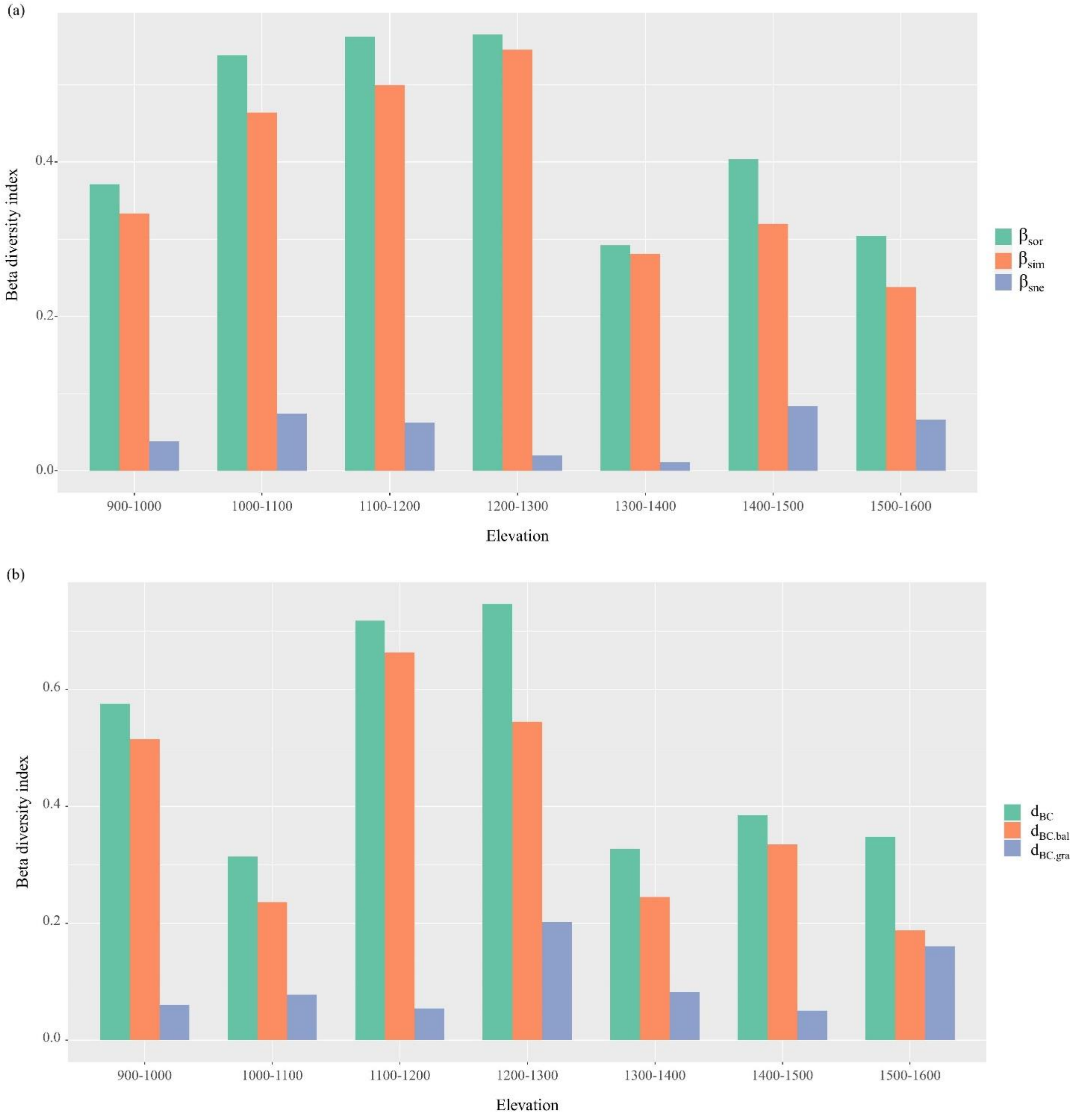

Supplemental Fig.S2 Community beta diversity and its components between neighboring community. (a) displayed the beta diversity(β_{sor}), species turnover (β_{sim}), and species nestedness (β_{sne}) using presence-absence data. (b) presented the Bray-Curtis distance (d_{BC}), balance variation ($d_{BC\text{-}bal}$), and abundance gradient ($d_{BC\text{-}gra}$) using abundance data.
