# Supplementary material for: Environmental heterogeneity determines beta diversity and species turnover for woody plants along an elevation gradient in subtropical forests of China
Source: For Res (Fayettev). 2023 Oct 31;3:26. doi: 10.48130/FR-2023-0026 (PMC11524245; doi:10.48130/FR-2023-0026)
Supplement: Supplementary file 1 — Supplementary data to this article can be found online. [file FR-2023-0026-S1.zip › 10.48130_FR-2023-0026-Suppl-TableS1.pdf]

Supplemental Tables S1 Model comparison of linear and polynomial regression. If the *P*<0.05, binomial regression is chosen; otherwise, linear regression is chosen. The designation ** signifies that *P*<0.01.

Data	Beta indices	Model	Res.Df	RSS.Df	Sum of Sq	*F*	*P*
Abundance	beta	Y~X	26	0.5222			
		Y~X+I(X^2)	25	0.37273	0.14948	10.026	0.004**
	turnover	Y~X	26	0.5475			
		Y~X+I(X^2)	25	0.3959	0.15161	9.5746	0.005**
	nestedness	Y~X	26	0.0499			
		Y~X+I(X^2)	25	0.0499	7.54E-06	0.0038	0.952
Presence-absence	beta	Y~X	26	0.2033			
		Y~X+I(X^2)	25	0.1889	0.014375	1.9023	0.18
	turnover	Y~X	26	0.3466			
		Y~X+I(X^2)	25	0.3249	0.021671	1.6673	0.208
	nestedness	Y~X	26	0.0528			
		Y~X+I(X^2)	25	0.0521	0.000746	0.3581	0.555
