# Supplementary material for: Environmental heterogeneity determines beta diversity and species turnover for woody plants along an elevation gradient in subtropical forests of China
Source: For Res (Fayettev). 2023 Oct 31;3:26. doi: 10.48130/FR-2023-0026 (PMC11524245; doi:10.48130/FR-2023-0026)
Supplement: Supplementary file 1 — Supplementary data to this article can be found online. [file FR-2023-0026-S1.zip › 10.48130_FR-2023-0026-Suppl-TableS2.pdf]

Supplemental Tables S2. Forward selection of explained variables

	AIC	*F*	*P*
+AT	-7.0678	5.9321	0.002**
+SLOP	-8.1669	2.3656	0.028*
+PCNM1	-8.5341	1.3773	0.220
+SPO	-7.8789	0.9545	0.476
+TP	-7.9089	0.9731	0.482
+PCNM2	-7.7593	0.8810	0.568
+pH	-7.5411	0.7496	0.648
+TC	-7.5697	0.7666	0.650
+PCNM4	-7.4053	0.6697	0.684
+SWC	-7.2871	0.6012	0.816
