# Supplementary material for: Environmental heterogeneity determines beta diversity and species turnover for woody plants along an elevation gradient in subtropical forests of China
Source: For Res (Fayettev). 2023 Oct 31;3:26. doi: 10.48130/FR-2023-0026 (PMC11524245; doi:10.48130/FR-2023-0026)
Supplement: Supplementary file 1 — Supplementary data to this article can be found online. [file FR-2023-0026-S1.zip › 10.48130_FR-2023-0026-Suppl-TableS3.pdf]

Supplemental Tables S3 Significance test of PCNM

	Df	Variance	*F*	*P*
Model	2	0.1524	0.9129	0.485
Residual	5	0.4174		

Model: rda(X = DYS.hel, Y = DYS.xy)

Permutation test for rda under reduced model

Number of permutations: 999
