# Supplementary material for: Environmental heterogeneity determines beta diversity and species turnover for woody plants along an elevation gradient in subtropical forests of China
Source: For Res (Fayettev). 2023 Oct 31;3:26. doi: 10.48130/FR-2023-0026 (PMC11524245; doi:10.48130/FR-2023-0026)
Supplement: Supplementary file 1 — Supplementary data to this article can be found online. [file FR-2023-0026-S1.zip › 10.48130_FR-2023-0026-Suppl-FigureS1.pdf]

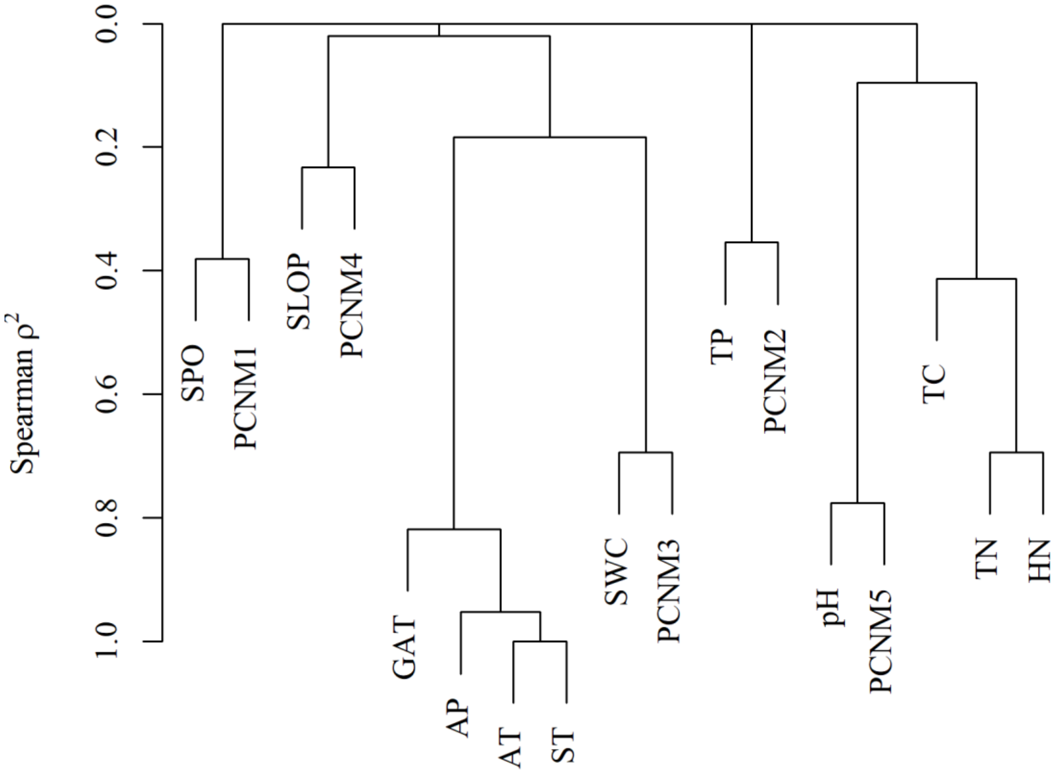

Supplemental Fig.S1 Multiple collinearity analysis of environmental variables. If Spearman $\rho^2 > 0.5$, it can be inferred that there is a strong collinearity between these factors, and one of them kept while the others removed. We retained SPO, PCNM1, SLOP, PCNM4, AT, SWC, TP, PCNM2, pH and TC.
